# Autocrine C‐peptide protects INS1 β cells against palmitic acid‐induced oxidative stress in peroxisomes by inducing catalase

**DOI:** 10.1002/edm2.147

**Published:** 2020-05-30

**Authors:** Patrizia Luppi, Nicholas Drain, Ramsey To, Donna Stolz, Callen Wallace, Simon Watkins, Peter Drain

**Affiliations:** ^1^ Department of Cell Biology University of Pittsburgh School of Medicine Pittsburgh PA USA

**Keywords:** apoptosis, autocrine, C‐peptide, diabetes, oxidative stress, palmitic acid, reactive oxygen species (ROS), β cells

## Abstract

**Aims:**

C‐peptide, produced by pancreatic β cells and co‐secreted in the bloodstream with insulin, has antioxidant properties in glucose‐ and hydrogen peroxide (H_2_O_2_)‐exposed INS1 β cells. Palmitic acid, the most physiologically abundant long‐chain free fatty acid in humans, is metabolized in peroxisomes of β cells accumulating H_2_O_2_ that can lead to oxidative stress. Here, we tested the hypothesis that C‐peptide protects β cells from palmitic acid‐induced stress by lowering peroxisomal H_2_O_2_.

**Materials and methods:**

We exposed INS1 β cells to palmitic acid and C‐peptide in the setting of increasing glucose concentration and tested for changes in parameters of stress and death. To study the ability of C‐peptide to lower peroxisomal H_2_O_2_, we engineered an INS1 β cell line stably expressing the peroxisomal‐targeted H_2_O_2_ sensor HyPer, whose fluorescence increases with cellular H_2_O_2_. An INS1 β cell line stably expressing a live‐cell fluorescent catalase reporter was used to detect changes in catalase gene expression.

**Results:**

C‐peptide protects INS1 β cells from the combined effect of palmitic acid and glucose by reducing peroxisomal H_2_O_2_ to baseline levels and increasing expression of catalase.

**Conclusions:**

In conditions of glucolipotoxicity, C‐peptide increases catalase expression and reduces peroxisomal oxidative stress and death of INS1 β cells. Maintenance of C‐peptide secretion is a pro‐survival requisite for β cells in adverse conditions. Loss of C‐peptide secretion would render β cells more vulnerable to stress and death leading to secretory dysfunction and diabetes.

## Graphical abstract

Proinsulin C‐peptide has antioxidant properties in glucose‐ and hydrogen peroxide (H_2_O_2_)‐exposed INS1 beta cells. Here, we tested the hypothesis that C‐peptide protects beta cells from palmitic acid‐induced stress by lowering peroxisomal H_2_O_2_. We exposed INS1 β cells to palmitic acid and C‐peptide in the setting of increasing glucose concentration and tested for changes in parameters of stress and death. To study the ability of C‐peptide to lower peroxisomal H_2_O_2_, we engineered an INS1 β cell line stably expressing the peroxisomal‐targeted H_2_O_2_ sensor HyPer, whose fluorescence increases with cellular H_2_O_2_. An INS1 beta cell line stably expressing a live‐cell fluorescent catalase reporter was used to detect changes in catalase gene expression. We found that in conditions of glucolipotoxicity, C‐peptide increases catalase expression and reduces peroxisomal oxidative stress and death of INS1 beta cells. We conclude that maintenance of C‐peptide secretion is a pro‐survival requisite for beta cells. Therefore, loss of C‐peptide secretion would render beta cells more vulnerable to stress leading to secretory dysfunction and diabetes.

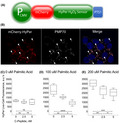

## 1 INTRODUCTION

Serum conditions associated with diabetes, such as elevation of glucose, saturated free fatty acids (FFAs) and inflammatory cytokines, elicit intracellular production of reactive oxygen species (ROS) generating oxidative stress, which is a leading factor triggering pancreatic β cell degeneration in diabetes. As a consequence, type 1 and type 2 diabetes (T1D and T2D) subjects suffer from variable degrees of loss of β cells and impaired β cell secretion of both insulin and C‐peptide.^1^, ^2^, ^3^, ^4^, ^5^, ^6^

C‐peptide is the 31 amino acid peptide generated in the secretory granules of pancreatic β cells as part of normal insulin biosynthesis.^7^ After its cleavage from proinsulin, C‐peptide is stored in the β cell secretory granules and co‐secreted in equimolar amount with insulin in the bloodstream of healthy individuals in response to ever‐changing glycaemia. However, C‐peptide does not undergo as much hepatic retention as insulin and circulates at a concentration approximately tenfold higher than that of insulin, with a biological half‐life of more than 30 minutes in healthy adult humans, compared to 3‐4 minutes for insulin.^8^, ^9^ Although for several decades C‐peptide has been thought to have no biological activity of its own, more recent evidence point to a role of C‐peptide as a ‘sensor‐effector’ of cellular stress able to directly reduce ROS generation by inhibiting glucose‐activated nicotinamide adenine dinucleotide phosphate (NADPH) oxidase at the plasma membrane^10^, ^11^ and restoring normal electron transport chain activity at mitochondria of endothelial cells.^12^, ^13^ In so doing, C‐peptide inhibits downstream deleterious effects associated with ROS accumulation and inhibits pro‐apoptosis enzymes caspase‐3 and transglutaminase‐2, while stimulating expression of survival protein Bcl‐2 in a variety of peripheral target cells.^10^, ^11^, ^14^, ^15^, ^16^ Our laboratory has demonstrated a novel C‐peptide mechanism, in which its beneficial activity expands to the same pancreatic β cells that synthesize and secrete C‐peptide, in an autocrine fashion.^17^ Thus, C‐peptide appears to be more than a coincidental ‘bystander’ and could be directly acting on β cells over time to maintain a healthy secretory status. Stressful conditions that compromise C‐peptide secretion might therefore put β cells at risk for further stress and death contributing to diabetes.^17^, ^18^

Palmitic acid (C16:0) is the most physiologically abundant long‐chain (LC) saturated FFA in the body and is elevated by industrialized diets.^19^ High circulating levels of FFAs are a common feature of T2D, particularly in overweight subjects,^20^, ^21^ and are also found in T1D patients.^22^ Prolonged exposure of β cells to LC (C10‐C16) and very LC (C17‐C24) FFAs inhibits glucose‐induced insulin secretion, increases intracellular ROS production, and triggers β cell death, in a phenomenon termed lipotoxicity.^19^, ^23^, ^24^, ^25^ The toxic actions of FFAs on β cells are dramatically increased in the setting of high glucose (glucolipotoxicity).^26^ An early event leading to β cell oxidative stress after palmitic acid exposure is an increase in H_2_O_2_ levels^27^ from β‐oxidation mainly at two subcellular sites, the mitochondria and the peroxisomes. However, excess of palmitic acid and very LC‐FFAs which are highly toxic to β cells, critically depend on peroxisomal detoxification for β cell survival.^28^

Peroxisomes are single membrane‐bound, complex, and highly dynamic organelles present virtually in every eukaryotic cell.^29^ Peroxisomes contain oxidase enzymes for FFA β‐oxidation, generating H_2_O_2_ as a by‐product in addition to shortened acyl‐CoA, and the enzyme catalase for H_2_O_2_ detoxification. While the fatty acyl‐CoA metabolites generated by β‐oxidation are imported into mitochondria for further oxidation, which generates ATP, the reactive H_2_O_2_ remains in the peroxisomes and if left to accumulate becomes toxic to cells. The detoxifying enzyme catalase protects the cells against the oxidative damage by decomposing H_2_O_2_ into water and molecular oxygen (O_2_). Peroxisomes are therefore specialized sites in the cell where LC‐ and very LC‐FFAs‐induced H_2_O_2_ is both generated and scavenged. Experiments in which catalase was either overexpressed in the peroxisome or in the mitochondria, showed that only peroxisomal catalase provided protection against LC‐FFAs‐induced lipotoxic death of β cells, while mitochondrial catalase was not protective, thus demonstrating that peroxisomally generated H_2_O_2_ mediates lipotoxicity in β cells.^28^ Catalase is present in peroxisomes of most tissue, but at a low basal level in peroxisomes of pancreatic β cells, which renders these cells exquisitely sensitive to abnormally high H_2_O_2_ levels.^30^, ^31^, ^32^

Based on these observations, and that the major toxic product of palmitic acid metabolism is H_2_O_2_ in the peroxisomes, we hypothesized that C‐peptide can protect β cells against palmitic acid‐induced stress by stimulating cellular pathways with antioxidant effectors in peroxisomes. While this hypothesis has remained untested generally for all cell types, in these studies we chose to test it specifically for β cells, which are the only cells of the body that make and secrete C‐peptide. A protective autocrine action of C‐peptide against palmitic acid‐induced β cell stress would provide amplified protection to other peripheral target cells by protecting the very β cells that make and secrete the protecting agent, C‐peptide, thereby providing more C‐peptide in a positive feedback loop.

## 2 MATERIALS AND METHODS

### 2.1 Cell culture of INS1 β cells

The rat insulinoma β cell line INS1 (clone 832/13) was a kind gift from Dr Chris Newgard.^33^ INS1 β cells were cultured in T‐75 cm^2^ flasks (Corning) in regular RPMI‐1640 medium (Gibco, Invitrogen) unless indicated otherwise, with 2 mmol/L L‐glutamine (Gibco, Invitrogen), 10% heat‐inactivated foetal bovine serum (Atlanta, Optima), 100 U/mL penicillin, 100 μg/mL streptomycin, 10 mmol/L HEPES, and 50 mmol/L β‐mercaptoethanol (all from Gibco, Invitrogen) at 37°C, 5% CO_2_. Cells were passaged once a week when reaching 90% confluence and used between passages 30 and 65. In certain experiments, cells were serum‐starved with minimum medium constituted by RPMI‐1640 medium (Gibco, Invitrogen), 2 mmol/L L‐glutamine (Gibco, Invitrogen), 1 mmol/L Sodium Pyruvate (Gibco, Invitrogen), 0.1% bovine serum albumin (BSA; fat‐free, Sigma), 100 U/mL penicillin, 100 μg/mL streptomycin, 10 mmol/L HEPES (Gibco, Invitrogen) and 3 mmol/L glucose. β‐mercaptoethanol was omitted in the medium in all conditions and assays of stress and death. A dose of 0‐10 nmol/L human C‐peptide (Phoenix Pharmaceuticals) was selected based on previous published reports.^10^, ^11^, ^12^, ^13^, ^15^, ^17^

### 2.2 Preparation of palmitic acid solution

Palmitic acid (Cayman Chemical Co.) was applied as a conjugate with BSA by dilution from 5 mmol/L palmitic acid/3.75% fatty acid‐free BSA stock, prepared as follows. A 20 mmol/L solution of palmitic acid was made in 8 mL of 0.01 mmol/L NaOH in double distilled water and heated to 70°C for 30 minutes to form a palmitic acid soap. The palmitic acid soap was added to 24 mL of 50 mg/L fatty acid‐free BSA (Sigma) in Phosphate Buffer Saline (PBS; Gibco) to form a stock solution of 5 mmol/L palmitic acid conjugated with 3.75% BSA. The 5 mmol/L palmitic acid/3.75% BSA stock was aliquoted and frozen at −20°C. For each experiment, one aliquot was thawed and diluted in the appropriate cell culture medium or PBS to the indicated palmitic acid concentration. For the 0 palmitate controls, the same procedure was used except that the stock was 0 mmol/L palmitic acid/3.75% fatty acid‐free BSA, which was diluted to the equivalent BSA concentration used in each experiment. After each use, thawed stock solutions were discarded.

### 2.3 Detection of cell death and apoptosis

INS1 β cells (10 000/well) were seeded in 96‐well plates in regular medium for 48 hours at 37°C, 5% CO_2_. Then, cells were serum‐starved overnight in minimum medium at 37°C, 5% CO_2_. The next day, medium was replaced with minimum medium with either 5.5, or 11, or 22 mmol/L glucose with or without 5 or 10 nmol/L C‐peptide (Phoenix Pharmaceuticals) for 20 minutes at 37°C, 5% CO_2_ before adding 100 or 200 μmol/L palmitic acid or equimolar BSA (Gibco) as control. Cells were then incubated for 24 hours at 37°C, 5% CO_2_. To detect cell death, 1 μg/mL of propidium iodide (PI; Sigma) was added and incubated in the dark on a rocking plate at room temperature for 15 minutes. PI is a red‐fluorescent nuclear and chromatin counterstain that is not permeant to live cells, therefore commonly used to detect dead cells in a population. PI fluorescence excitation was 535 nm and emission was read at 617 nm using a computer‐controlled BioTek Synergy H1 Hybrid Multi‐Mode plate reader (Winooski, VT). Each condition was tested in a total of 24 independent experiments. Cell death is shown as arbitrary units of PI fluorescence at 617 nm expressed as mean ± standard error of the mean (SEM).

To detect INS1 β cell apoptosis, cells (10 000/well) were seeded in 48‐well plates in regular medium for 72 hours at 37°C, 5% CO_2_. Then, cells were serum‐starved overnight in minimum medium at 37°C, 5% CO_2_. The next day, medium was replaced with minimum medium with 11 mmol/L glucose with or without 5 or 10 nmol/L C‐peptide (Phoenix Pharmaceuticals) for 20 minutes at 37°C, 5% CO_2_ before adding 200 μmol/L palmitic acid or equimolar BSA (Gibco) as control. Cells were then incubated for 24 hours at 37°C, 5% CO_2_. Apoptosis was detected using the Cell Death Detection ELISA^PLUS^ kit (Roche Diagnostics Mannheim) following manufacturer's instructions. Each condition was tested in 18 independent experiments. Apoptosis is shown as the difference in units of absorbance at 405 and 490 nm and expressed as mean ± SEM

### 2.4 Generation of INS1 β cells stably expressing the mCherry‐HyPer‐PTS1 biosensor

In order to test whether C‐peptide lowers peroxisomal H_2_O_2_, we sought a method that measures H_2_O_2_ levels specifically in peroxisomes. This process has been well‐studied in β cells by Elsner *et al* ^28^ who used the genetically encoded HyPer, a green fluorescent protein whose fluorescence increases with cellular H_2_O_2_ over the μmol/L range, with a C‐terminal PTS1 signal sequence for import into peroxisomes. Following their lead, we constructed a mammalian expression plasmid pAAN11_mCherry‐HyPer‐PTS1. Briefly, the two Kas1 sites of the Puro gene within pRNA‐CMV3.1/Puro (Genscript) were removed and its polylinker replaced using BamHI and XbaI sites by the new polylinker with top sequence GGATCCACCGGTCGCCACCATGGCGGC CGCTAGCTCGAGTCAGAGCTCAGGTGCGAATTCATCATAAGCTTAATAATAAAGGATCTTTTATTTTCATTGGATCTGTGTGTTGGTTTTTTGTGTGGGGCCGCCCTCGACTGTGCCTTCTAGA. By using NotI and KasI the fluorescent protein mCherry^34^ was inserted downstream from the CMV promoter so that we could easily normalize the HyPer fluorescence, which varies according to the H_2_O_2_ levels, to a constant mCherry fluorescence localized in peroxisomes. Finally, by using KasI and HindIII, HyPer‐PTS1 was inserted fused in‐frame downstream from mCherry. The PTS1 sequence is TCCAAGCTGtga and encodes the C‐terminus of the fusion protein SerLysLeuStop.

We then established a INS1 β cell line expressing the mCherry‐HyPer‐PTS1 biosensor as approximately 100 mCherry fluorescent puncta per cell. Briefly, INS1 β cells were seeded in six‐well plates overnight and the next day transfected with pAAN11_mCherry‐HyPer‐PTS1 by using Lipofectamine (Invitrogen). The next day, selection of transfected cells was started with 2 μg/mL puromycin. After 2 weeks of puromycin selection, positive cells were transferred to separate flasks, grown and tested for co‐localization of mCherry fluorescence with the peroxisomal marker PMP70 by immunofluorescence (see below). One of the cell lines showing >90% co‐localization was designated mCherry‐HyPer‐PTS1‐2 and used for all further experiments.

### 2.5 Immunofluorescence to assay co‐localization of mCherry‐PTS1 with PMP70 peroxisomal marker

INS1 β cells stably expressing the mCherry‐HyPer‐PTS1 biosensor were grown on coverslips and fixed in 2% paraformaldehyde in PBS (Gibco, Invitrogen) for 1 hour Cells were washed three times for 5 minutes with PBS (Gibco, Invitrogen), then permeabilized for 20 minutes in 0.1% Triton X‐100 in PBS (Gibco, Invitrogen). After washing three times for 5 minutes with BSA solution (0.5% [w/v] BSA, 0.15% [w/v] glycine in PBS), cells were blocked with 20% (v/v) normal goat serum in BSA solution for 40 minutes. Following three washes, cells were incubated with a rabbit anti‐PMP70 antibody (Santa Cruz, Biotechnology; 1:100) for 1 hour at room temperature. After washing three times with BSA solution, sections were incubated for 1 hour with a goat anti‐rabbit Alexa 488 secondary antibodies (Invitrogen, 1:500) before being washed three times with BSA solution, then once with Hoechst dye (1 g/100 mL) for 30 seconds to stain nuclear DNA. The sections were washed and placed on coverslips with Gelvatol, a water‐soluble mounting medium. Cells were evaluated using an Olympus Fluoview 1000 confocal microscope.

### 2.6 Determination of palmitic acid‐induced ROS in peroxisomes of mCherry‐HyPer‐INS1 β cells

Stably transfected mCherry‐HyPer‐PTS1‐2 INS1 β cells (50 000/well) were seeded in 24‐well plates in regular medium and kept at 37°C, 5% CO_2_ for 48 hours. Medium was removed and replaced with PBS with 11 mmol/L glucose with or without 2.5 or 5 nmol/L C‐peptide (Phoenix Pharmaceuticals) for 20 minutes at 37°C, 5% CO_2_ before adding 100 or 200 μmol/L palmitic acid for 30 minutes at 37°C, 5% CO_2_. Cells exposed to equimolar amounts of BSA were used as a control. Generation of H_2_O_2_ was assayed by immediately measuring fluorescence of HyPer (excitation 488; emission 510 nm) and of mCherry (excitation at 587; emission at 610) in each well using the BioTek Synergy H1 Hybrid Multi‐Mode plate reader. Each condition was tested in a total of 18 independent experiments. Hyper fluorescence was normalized to the mCherry fluorescence in each well and expressed as Hyper‐live‐cell fluorescence arbitrary units mean ± SEM

### 2.7 Live‐cell catalase expression assays using CAT minigene‐mNeonGreen reporter

To test for changes in catalase gene expression, we used a rat catalase minigene with DNA sequences encoding mNeonGreen fused to the C‐terminal sequences encoding the catalase protein. The reporter includes 1874 bases of the catalase gene promoter including its PPARγ enhancer and the native 5′UTR and the entire catalase protein coding sequences followed by the mNeonGreen sequences. Stable transfectants of the reporter in the INS1 β cells were isolated using a vector puromycin resistance marker, as described for the mCherry‐Hyper INS1 β cell line above.

Once generated, INS1 β cells stably expressing the catalase reporter were seeded in 24‐well plates (50 000/well) in regular medium and kept at 37°C, 5% CO_2_ for 48 hours. Medium was removed and replaced with PBS with 11 mmol/L glucose in the absence or presence of 5, 10 nmol/L or 10 nmol/L heat‐inactivated C‐peptide (Phoenix Pharmaceuticals) for 20 minutes at 37°C, 5% CO_2_ before addition of 200 μmol/L palmitic acid at 37°C, 5% CO_2_ for 24 hours. C‐peptide was heat‐inactivated by boiling it for 1 hour and quickly chilled in ice immediately before addition to cells in culture.^35^ Cells exposed to equimolar amounts of BSA were used as a 0 μmol/L palmitic acid control. Each condition was tested in a total of 20 independent experiments performed over a two‐month period. In each experiment changes in normalized green fluorescence were measured and normalized to Hoechst to account for any cell number variability from well to well by using the BioTek Synergy H1 Hybrid Multi‐Mode plate reader, and expressed as live‐cell fluorescence arbitrary units mean ± SEM

### 2.8 Statistical analysis

Results are presented in boxplots, with the box indicating the central 50% of the data in which a line indicates the median and the whiskers the range. ANOVA followed by the Tukey post hoc test was used to assess differences between different conditions using Prism 8 (GraphPad Software, Inc). Values of *P* < .05 were considered statistically significant.

## 3 RESULTS

### 3.1 Glucose enhances palmitic acid‐induced INS1 β cell death

**Figure 1:**
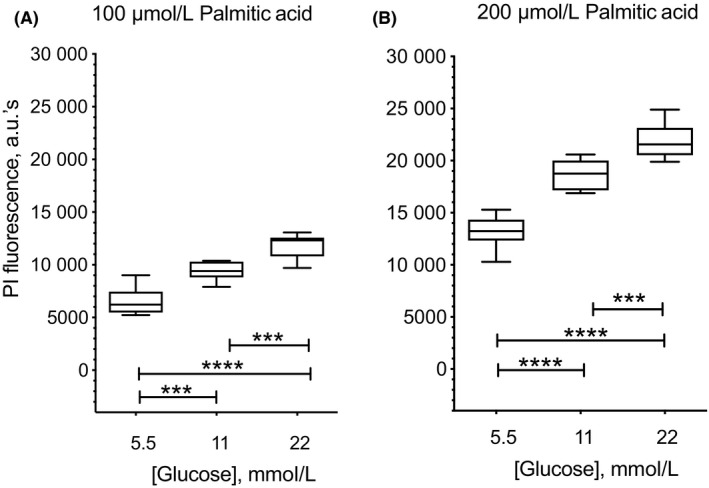
Glucose enhances lipotoxic effect of palmitic acid in INS1 β cells. Cells were seeded in 96‐well plate and cultured in minimum medium with either 100 μmol/L (panel A) or 200 μmol/L (panel B) palmitic acid in the presence of either 5.5, 11, or 22 mmol/L glucose for 24 h at 37°C, 5% CO_2_. To detect cell death, 1 μg/mL propidium iodide (PI) was added to each well and fluorescence detected after 15 min incubation using a BioTek H1 Hybrid plate reader. INS1 β cell death is shown as arbitrary units of PI fluorescence at 617 nm. In panel A, cell death induced by 100 μmol/L palmitic acid increased from 5.5 to 11 mmol/L glucose (***P < .005), with the highest value detected with 22 mmol/L glucose as compared to both 5.5 mmol/L (****P < .0001) and 11 mmol/L (***P < .001) glucose concentration. Similarly, in panel B, cell death induced by 200 μmol/L palmitic acid increased from 5.5 to 11 mmol/L glucose (****P < .0001), with the highest value detected with 22 mmol/L glucose as compared to both 5.5 mmol/L (****P < .0001) and 11 mmol/L (***P < .005) glucose concentration. Increasing palmitic acid concentration from 100 to 200 μmol/L also significantly increased cell death at any given glucose concentration (*P* < .0001). Results are presented in boxplots, with the box indicating the central 50% of the data in which a line indicates the median and the whiskers the range

Exposure of β cells to elevated levels of palmitic acid causes oxidative stress and death. The toxic effect of palmitic acid on β cells is enhanced by concomitant elevated glucose (glucolipotoxicity). We therefore sought to study the effect of increasing glucose on palmitic acid‐induced INS1 β cell death as measured by PI fluorescence. In Figure 1 (panel A), cell death induced by 100 μmol/L palmitic acid increased from 5.5 mmol/L (6519 ± 453.9 arbitrary units of PI fluorescence) to 11 mmol/L glucose (9407 ± 305.9) (*P* < .005), with the highest value detected with 22 mmol/L glucose (11 802 ± 411.8) as compared to both 5.5 mmol/L (*P* < .0001) and 11 mmol/L (*P* < .005). Similarly, as shown in panel B, cell death induced by 200 μmol/L palmitic acid increased from 5.5 mmol/L (13 136 ± 539.3) to 11 mmol/L glucose (18 675 ± 534.9; *P* < .0001), with the highest value detected with 22 mmol/L glucose (21 910 ± 592.9) as compared to both 5.5 mmol/L (*P* < .0001) and 11 mmol/L glucose (*P* < .005). Increasing palmitic acid concentration from 100 to 200 μmol/L also significantly increased INS1 β cell death at any given glucose concentration (*P* < .0001).

### 3.2 C‐peptide protects INS1 β cells from palmitic acid‐induced cell death

**Figure 2:**
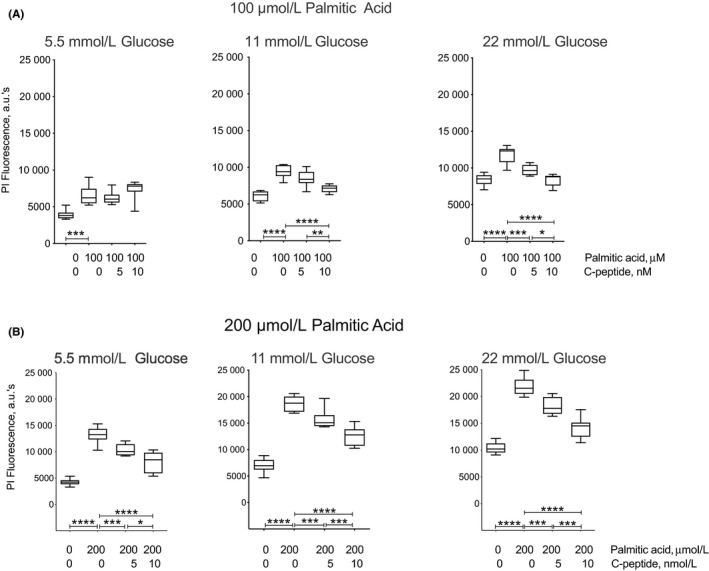
C‐peptide decreases palmitic acid‐induced INS1 β cell death. Cells were plated in 96‐well plates in regular medium and seeded for 48 h at 37°C, 5% CO_2_. Then, they were serum‐starved for 24 h in minimum medium at 37°C, 5% CO_2_. The next day, medium was replaced with minimum medium with either 5.5, or 11, or 22 mmol/L glucose in the absence or presence of 5 or 10 nmol/L C‐peptide for 20 min at 37°C, 5% CO_2_ before addition of 100 or 200 μmol/L palmitic acid, or equimolar Bovine Serum Albumin (BSA) as 0 palmitic acid control. Cells were then incubated for 24 h at 37°C, 5% CO_2_. To detect cell death, 1 μg/ml of propidium iodide (PI) was added to each well, and fluorescence assayed after 15 min incubation using a BioTek H1 Hybrid plate reader. INS1 β cell death is shown as arbitrary units of PI fluorescence at 617 nm. In panel A, 100 μmol/L palmitic acid‐induced an increased cell death compared to BSA in 5.5 mmol/L (***P < .005), 11 mmol/L (****P < .0001), and 22 mmol/L glucose (****P < .0001). Addition of 5 nmol/L C‐peptide decreased palmitic acid‐induced cell death in the presence of 22 mmol/L glucose (***P < .005). A further reduction in cell death was observed with 10 nmol/L C‐peptide in 11 mmol/L (****P < .0001) and 22 mmol/L glucose (****P < .0001), and this reduction was significant when compared to 5 nmol/L C‐peptide in both 11 mmol/L (**P < .01) and 22 mmol/L glucose (*P < .05). While palmitic acid significantly increased cell death compared to BSA alone (***P < .005) in 5.5 mmol/L glucose, C‐peptide addition did not show a significant reduction in cell death at this glucose concentration. The protective effect of C‐peptide on palmitic acid‐induced beta cell death extends to conditions of 200 μmol/L palmitate exposure, as shown in panel B. Palmitic acid‐induced cell death increased in all glucose concentrations as compared to BSA control (*P* < .0001). Addition of 5 nmol/L C‐peptide protected against palmitic acid‐induced cell death in all glucose concentrations compared to palmitic acid alone (***P < .005). A further protection was achieved with 10 nmol/L C‐peptide as compared to palmitic acid alone in all glucose concentrations (****P < .0001), and this protection was significantly better than the one achieved with 5 nmol/L C‐peptide in 5.5 mmol/L (*P < .05), 11 and 22 mmol/L glucose (***P < .005). Results are presented in boxplots, with the box indicating the central 50% of the data in which a line indicates the median and the whiskers the range

We next analysed the effect of C‐peptide on palmitic acid‐induced INS1 β cell death in the broad setting of varying glucose concentrations. Figure 2 (Panel A) shows that in condition of exposure to 100 μmol/L palmitic acid, 5 nmol/L C‐peptide tended to reduce cell death at all glucose concentrations, reaching significance only at 22 mmol/L glucose (9702 ± 244.2 arbitrary units of PI fluorescence) as compared to palmitic acid alone (11 802 ± 411.8; *P* < .005). Addition of 10 nmol/L C‐peptide completely reversed palmitic acid‐induced cell death to levels detected in the BSA control in 11 mmol/L glucose (7083.9 ± 180.6) as compared to palmitic acid (9407 ± 305.9; *P* < .0001), and in 22 mmol/L glucose (8364 ± 288.2) as compared to palmitic acid (11 802 ± 411.8; *P* < .0001). The cytoprotective effect achieved with 10 nmol/L C‐peptide was significant when compared to the effect with 5 nmol/L C‐peptide in the presence of both 11 mmol/L (*P* < .01) and 22 mmol/L glucose (*P* < .05), suggesting the presence of a C‐peptide dose‐response effect.

The protective effect of C‐peptide on palmitic acid‐induced INS1 β cell death extends to conditions in which its concentration was increased to 200 μmol/L, as shown in Figure 2 (panel B). Addition of 5 nmol/L C‐peptide protected against palmitic acid‐induced cell death in all glucose concentrations, with values of 10 368 ± 391.0 in 5.5 mmol/L glucose, values of 15 674 ± 638.9 in 11 mmol/L glucose, and 18 206 ± 555.5 in 22 mmol/L glucose compared to palmitic acid alone (*P* < .005). A further decrease in palmitic acid‐induced cell death to levels close to those measured with the BSA control, was detected with 10 nmol/L C‐peptide as compared to palmitic acid alone, with values of 8026.5 ± 685.3 in 5.5 mmol/L glucose, 12 546 ± 646.0 in 11 mmol/L glucose, and 14 244 ± 675.4 in 22 mmol/L glucose (*P* < .0001). Again, the cytoprotective effect achieved with 10 nmol/L C‐peptide was significantly better than 5 nmol/L C‐peptide in all glucose concentrations (in 5.5 mmol/L *P* < .05 and 11 and 22 mmol/L glucose *P* < .005), demonstrating the presence of a C‐peptide dose‐response effect. Whereas the effects of 100 and 200 μmol/L palmitic acid elevated cell death, and the protecting effects of C‐peptide protecting held true qualitatively at all three glucose concentrations, increasing glucose concentration increased the differences in the magnitude of both the palmitic acid and C‐peptide effects.

### 3.3 C‐peptide protects INS1 β cells from palmitic acid‐induced death by decreasing apoptosis

**Figure 3:**
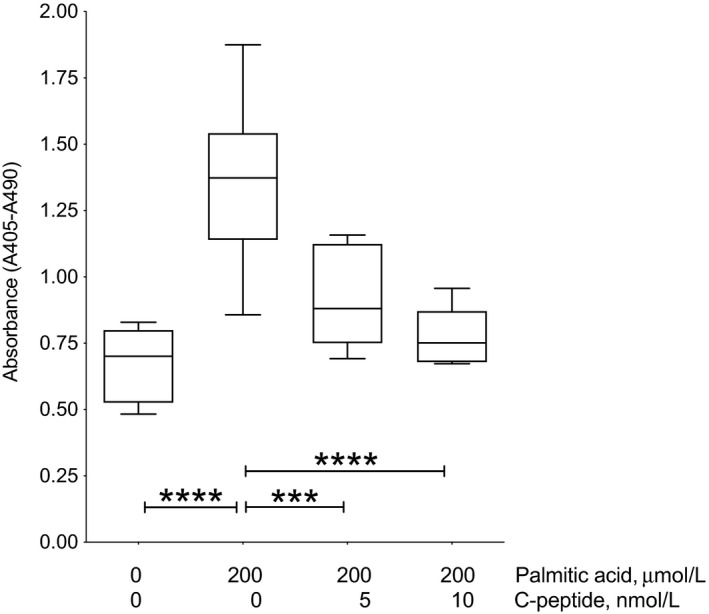
C‐peptide decreases palmitic acid‐induced INS1 β cell apoptosis. To detect apoptosis, cells were plated in 48‐well plates in regular medium and seeded for 72 h at 37°C, 5% CO_2_. Then, cells were serum‐starved for 24 h in minimum medium. The next day, medium was replaced with minimum medium with 11 mmol/L glucose in the absence or presence of 5 or 10 nmol/L C‐peptide for 20 min at 37°C, 5% CO_2_ before addition of 200 μmol/L palmitate or equimolar bovine serum albumin (BSA) as 0 palmitic acid control. Cells were then incubated for 24 h at 37°C, 5% CO_2_. Apoptosis was detected using the Cell Death Detection ELISA^PLUS^ kit (Roche Diagnostics Mannheim). Apoptosis is shown as the difference in units of absorbance at 405 and 490 nm. Addition of 200 μmol/L palmitic acid increased apoptosis as compared to control medium (****P < .0001). Addition of 5 nmol/L C‐peptide reduced apoptosis compared to cells in palmitic acid alone (***P < .005). Increasing C‐peptide from 5 to 10 nmol/L caused a further decrease in apoptosis compared to palmitic acid alone (****P < .0001). Results are presented in boxplots, with the box indicating the central 50% of the data in which a line indicates the median and the whiskers the range

To further investigate the effect of C‐peptide on palmitic acid‐induced INS1 β cell death with respect to the mechanisms involved, we exposed the cells to 200 μmol/L palmitic acid and tested the ability of C‐peptide to protect from apoptosis. Figure 3 shows that addition of 200 μmol/L palmitic acid for 24 hours increased apoptosis (1.356 ± 0.1 units of absorbance), as compared to the BSA control medium (0.674 ± 0.0; *P* < .0001). Addition of 5 nmol/L C‐peptide significantly reduced apoptosis (0.919 ± 0.0), compared to cells in palmitic acid alone (1.356 ± 0.1; *P* < .005). Increasing C‐peptide concentration to 10 nmol/L reversed palmitic acid‐induced apoptosis (0.778 ± 0.0; *P* < .0001) to levels close to baseline in BSA, however this decrease was not significantly different than the protection achieved with 5 nmol/L C‐peptide. These results indicate that in palmitic acid‐exposed INS1 β cells as well, C‐peptide displays cytoprotective activity at least in part through an inhibition of apoptotic pathways.

### 3.4 C‐peptide decreases palmitic acid‐induced H_2_O_2_ levels in peroxisomes of INS1 β cells

**Figure 4:**
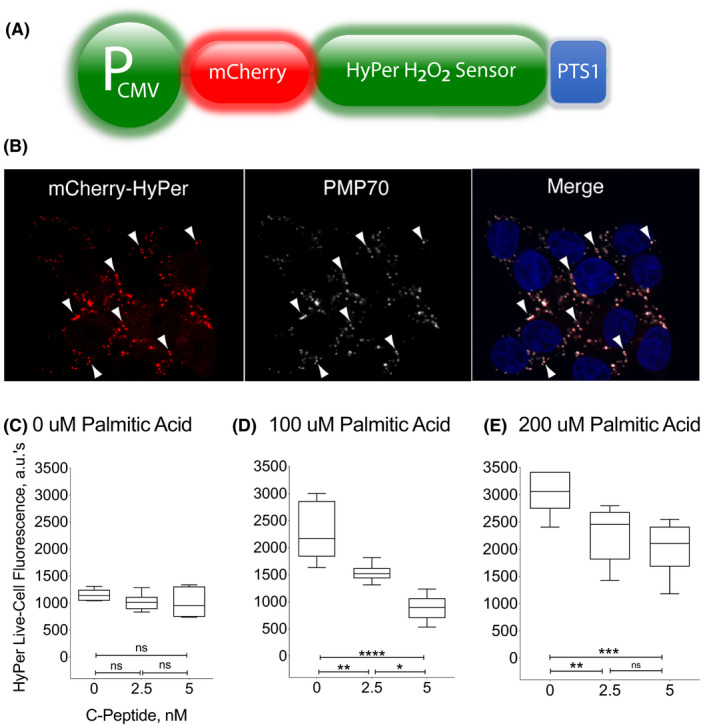
C‐peptide reduces palmitic acid‐induced H_2_O_2_ in peroxisomes of INS1 β cells. Panel A. Construction of a ratiometric peroxisomal H_2_O_2_ sensor. In order to test whether C‐peptide lowers peroxisomal H_2_O_2_ in INS1 β cells, we engineered a cell line stably expressing the peroxisomal‐targeted H_2_O_2_ biosensor mCherry‐HyPer‐PTS1, shown schematically here. See also Materials and Methods. Panel B. Localization of a ratiometric peroxisomal H_2_O_2_ sensor. Panel shows the mCherry‐Hyper as red fluorescence (see arrows), the peroxisomes showed as PMP‐70 positive green staining (see arrows), and the co‐localization of mCherry‐HyPer‐PTS1 with PMP‐70 is shown as yellow fluorescence puncta (merge; see arrows). Nuclear DNA is stained with Hoeschts dye and shown in blue. Cells were evaluated using an Olympus Fluoview 1000 confocal microscope. Panel C‐E, mCherry‐HyPer‐PTS1 INS1 β cells response to palmitic acid. Stably transfected cells were incubated with either 0, or 100, or 200 μmol/L palmitic acid for 30 min with 0, 2.5, and 5 nmol/L C‐peptide. After exposure to 100 and 200 μmol/L palmitic acid there was a dose‐dependent increase in peroxisomal H_2_O_2_, detected as increase in Hyper fluorescence as compared to 0 palmitic acid/BSA (*P* < .0001). The difference in palmitic acid‐induced H_2_O_2_ levels between 100 and 200 μmol/L was significant (*P* < .01). Addition of 2.5 and 5 nmol/L C‐peptide had no effect on H_2_O_2_ levels in 0 palmitic acid. However, in conditions of 100 μmol/L palmitic acid, addition of 2.5 and 5 nmol/L C‐peptide completely reversed peroxisomal H_2_O_2_ to baseline levels (**P < .01 and ****P < .0001, respectively). The reduction in peroxisomal H_2_O_2_ levels achieved by 5 nmol/L C‐peptide was different than with 2.5 nmol/L (*P < .05). We observed that 2.5 nmol/L C‐peptide decreased peroxisomal H_2_O_2_ levels in conditions of 200 μmol/L palmitic acid (**P < .01), and a further reduction was observed with 5 nmol/L C‐peptide (***P < .005), as compared to palmitic acid only. Results are presented in boxplots, with the box indicating the central 50% of the data in which a line indicates the median and the whiskers the range

Excessive β‐oxidation of LC‐FFAs causes cell death by excessive H_2_O_2_‐generation in peroxisomes of eukaryotic cells. We generated a INS1 β cell line stably expressing an mCherry‐Hyper biosensor (Figure 4; panel A) in the peroxisomes to study whether C‐peptide lowers H_2_O_2_ in peroxisomes after palmitic acid exposure. We validated the targeting of mCherry‐HyPer‐PTS1 to peroxisomes by measuring co‐localization of mCherry fluorescent puncta with the specific peroxisomal antigen PMP70 by immunohistochemistry (panel B). mCherry‐HyPer‐PTS1 fluorescence in red (see arrows) and the peroxisomes showed as PMP‐70 positive staining in green (see arrows) co‐localized, observed by the overlapping of mCherry‐HyPer‐PTS1 with PMP‐70 in yellow (Merge, see arrows). mCherry‐HyPer‐PTS1 is highly co‐localized with peroxisomes as shown in the yellow puncta. The fraction of mCherry puncta co‐localized with PMP‐70 was 0.95 (over 11 000 puncta across 200 cells).

Figure 4 also shows results with HyPer fluorescence of mCherry‐HyPer‐PTS1‐2 INS1 cells incubated with either 0 (panel C), or 100 (panel D), or 200 μmol/L palmitic acid (panel E). For each of these conditions, the cells were treated with 0, 2.5, and 5 nmol/L C‐peptide. The results show that 100 and 200 μmol/L palmitic acid exposure for 30 minutes generated a dose‐dependent increase in peroxisomal H_2_O_2_, detected as an increase in Hyper fluorescence from 2282.2 ± 215.4 arbitrary units to 3037 ± 155.6 arbitrary units, respectively, as compared to conditions in which no palmitic acid but only BSA was present (1148.5 ± 42.4; *P* < .0001; panel C). The difference of peroxisomal palmitic acid‐induced H_2_O_2_ levels between 100 and 200 μmol/L was significant (*P* < .01). Addition of 2.5 and 5 nmol/L C‐peptide had no effect on H_2_O_2_ levels in 0 μmol/L palmitic acid. However, in conditions of 100 μmol/L palmitic acid, addition of 2.5 and 5 nmol/L C‐peptide completely reversed peroxisomal H_2_O_2_ to baseline levels (1533.7 ± 66.5 *P* < .01 and 887.33 ± 97.0; *P* < .0001, respectively). The reduction in peroxisomal H_2_O_2_ levels achieved by 5 nmol/L C‐peptide was significantly different than that achieved with 2.5 nmol/L (*P* < .05), reflecting a dose‐dependent effect of C‐peptide.

C‐peptide also decreased peroxisomal H_2_O_2_ levels in conditions of 200 μmol/L palmitic acid with levels of 2287.2 ± 210.6 with 2.5 nmol/L C‐peptide (*P* < .01), and levels of 2026.8 ± 200 with 5 nmol/L C‐peptide (*P* < .005), as compared to palmitic acid only. The decrease in H_2_O_2_ levels achieved by 5 nmol/L C‐peptide was greater than the decrease obtained with 2.5 nmol/L C‐peptide, although the difference did not reach statistical significance difference. These results clearly indicate that C‐peptide is activating pathways that lower peroxisomal H_2_O_2_ levels elevated by palmitic acid.

### 3.5 Palmitic acid and C‐peptide stimulate catalase expression in INS1 β cells

**Figure 5:**
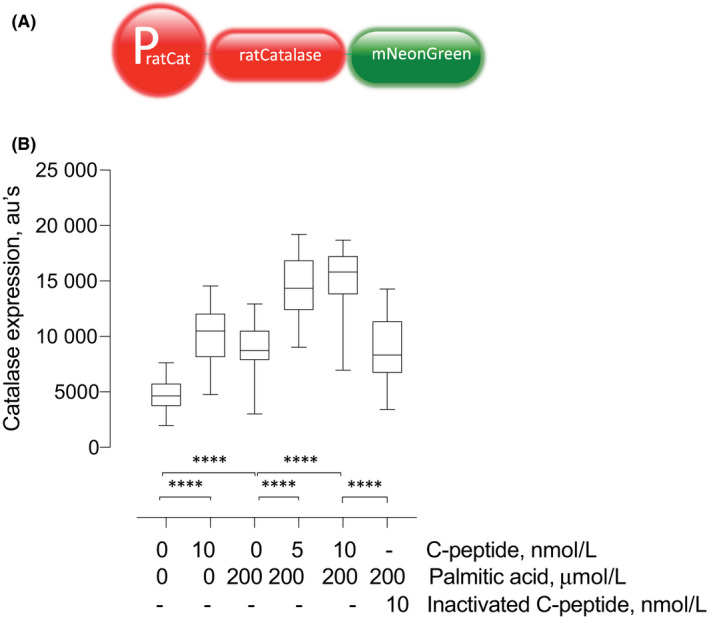
C‐peptide and palmitic acid stimulate catalase expression in INS1 β cells. Panel A, Construction of a CAT minigene‐mNeonGreen reporter. To test for changes in catalase gene expression, we used a rat catalase minigene with DNA sequences encoding mNeonGreen fused to the C‐terminal sequences encoding the catalase protein shown schematically here. See also Section 2. Panel B. Live‐cell catalase expression assays using CAT minigene‐mNeonGreen reporter. Cells were seeded in 24‐well plates in regular medium for 24 h at 37°C and 5% CO_2_ and then exposed to 200 μmol/L palmitic acid and 0, 5, 10 nmol/L C‐peptide or 10 nmol/L inactivated C‐peptide for 24 hr and assessed for catalase expression through fluorescence of the CAT minigene‐mNeonGreen reporter using a BioTek H1 Hybrid plate reader. Fluorescence was obtained with an excitation and emission wavelength of 490 and 520 nm, respectively. 200 μmol/L palmitic acid and 10 nmol/L C‐peptide both increased catalase expression (arbitrary units) compared to the control of no treatment (****P < .0001). Catalase expression increased with 200 μmol/L palmitic acid and 5 nmol/L C‐peptide (****P < .0001) and 10 nmol/L C‐peptide (****P < .0001) compared with 0 nmol/L C‐peptide. No significant difference was observed between 0 nmol/L C‐peptide and 10 nmol/L inactivated C‐peptide, but 10 nmol/L inactivated C‐peptide was significantly lower than 10 nmol/L C‐peptide (****P < .0001). Results are presented in boxplots, with the box indicating the central 50% of the data in which a line indicates the median and the whiskers the range

To further evaluate the mechanism by which C‐peptide lowers palmitic acid‐induced peroxisomal H_2_O_2_, we tested whether C‐peptide and palmitic acid increase peroxisomal catalase expression. We used a rat catalase minigene‐mNeonGreen reporter to measure the effects of C‐peptide and palmitic acid on catalase expression. Figure 5 shows fluorescence of the rat catalase‐mNeonGreen reporter in INS1 β cells exposed to 11 mmol/L glucose in the presence of 200 μmol/L palmitic acid and 0, 5, 10 nmol/L C‐peptide or 10 nmol/L inactivated C‐peptide. As shown, palmitic acid and C‐peptide each increased catalase expression independently, 10 nmol/L C‐peptide (10 129 ± 557.4 fluorescence arbitrary units) and 200 μmol/L palmitic acid (8802 ± 476.6) were each significantly higher than the control of no treatment (4829 ± 352.7; *P* < .0001). Furthermore, the presence of both palmitic acid and C‐peptide have an additive effect relative to either alone, with the highest expression detected with 200 μmol/L palmitic acid and 5 nmol/L (14 475 ± 635.3) or 10 nmol/L (15 114 ± 681.5) C‐peptide compared with either 10 nmol/L C‐peptide (10 129 ± 557.4) or 200 μmol/L palmitic acid alone (8802 ± 476.6; *P* < .0001). The expression of catalase‐mNeonGreen fluorescence in 200 μmol/L palmitic acid with 10 nmol/L inactivated C‐peptide (8886 ± 614.4) showed comparable levels to the level in 200 μmol/L palmitic acid with 0 nmol/L C‐peptide (8802 ± 476.6) and were significantly lower than in 0 μmol/L palmitic acid with 10 nmol/L C‐peptide (10 129 ± 557.4), indicating that a functional C‐peptide is responsible for the increase in catalase expression. These results indicate C‐peptide and palmitic acid increase catalase expression and that C‐peptide has autocrine protective capabilities through a catalase mechanism which is enhanced with palmitic acid.

## 4 DISCUSSION

The results reported in this study extend the concept of an autocrine antioxidant role of C‐peptide, in which INS1 β cells can adapt to high glucose and elevated palmitic acid exposure that are commonly associated with diabetes. In agreement with previous studies, we observed that palmitic acid‐induced INS1 β cell death was potentiated by a concomitant elevation of glucose in the culture medium (glucolipotoxicity),^26^ with the highest effect at the highest glucose concentration of 22 mmol/L. C‐peptide was able to protect cells from palmitic acid‐induced toxicity with a complete reversal of cell death in conditions of elevated glucose and/or palmitic acid in the culture medium, demonstrating that in the context of glucolipotoxicity as well, C‐peptide cytoprotective activity is largely stress‐dependent.^10^, ^13^, ^17^, ^18^, ^35^ C‐peptide displays little or no beneficial activity in basal cellular conditions or in cells exposed to a low level of stress. In fact, at the lowest glucose concentration of 5.5 mmol/L in the medium, C‐peptide showed pro‐survival activity at the highest palmitic acid concentration tested, suggesting that a certain threshold of cellular stress has to be reached to induce C‐peptide anti‐inflammatory pathways. These results suggest that C‐peptide acts as a sensor of cellular stress and activates effective adaptive responses when the accumulated level of stress becomes dangerous to cellular functions thereby preventing the generation of an irreversible and progressive state of stress that otherwise would be deleterious. Similar to what was observed in other C‐peptide sensitive cells, such as neurons, endothelial and smooth vascular cells, and kidney cells, the cytoprotective activity of C‐peptide in palmitic acid‐exposed INS1 β cells involves attenuation of apoptosis.^14^, ^15^, ^16^, ^17^, ^18^

Glucolipotoxicity is characterized by abnormally high levels of intracellular ROS leading to β cell apoptosis.^30^, ^31^, ^32^ Powerful adaptive responses, such as activation of antioxidant enzymes and anti‐inflammatory pathways, can lower ROS levels to recover β cell function. A state of chronic oxidative stress results from a persistent imbalance between an excessive production of ROS and a limited capacity of adaptive responses to detoxify these intermediates culminating in β cell secretory dysfunction and death. We have previously reported on the ability of C‐peptide to lower intracellular H_2_O_2_ levels in palmitic acid‐exposed INS1 β cells by using the green fluorescent dye 5‐(and‐6)‐chloromethyl‐2′‐7′‐dichlorodihydro‐fluorescein diacetate, acetyl ester (CM‐H_2_‐DCFDA), whose fluorescence intensity increases with increasing intracellular H_2_O_2_ levels.^24^

The current results corroborate these initial findings and expand on the cell biology by identifying peroxisomes as a novel organellar site of the antioxidant effects of C‐peptide. We focused on peroxisomes as they are the organelles where catabolism of excess of palmitic acid occurs by using stably transfected INS1 β cells expressing the mCherry‐HyPer‐PTS1 biosensor, whose fluorescence varies according to the H_2_O_2_ levels in peroxisomes.^28^ In a dose‐dependent fashion, C‐peptide reduced palmitic acid‐induced H_2_O_2_ to baseline levels detected in the absence of palmitic acid in peroxisomes of INS1 β cells. The effect was significant at the lowest C‐peptide concentration tested (2.5 nmol/L), and reached a complete reversal of the induced level of H_2_O_2_ with 5 nmol/L C‐peptide. In the absence of palmitic acid, addition of C‐peptide did not have any significant effects on baseline peroxisomal H_2_O_2_ showing that effect of C‐peptide was specific to excess palmitic acid‐induced H_2_O_2_.

The ability of C‐peptide to stimulate effector pathways specifically in peroxisomes of INS1 β cells that lower H_2_O_2_ is the single most important finding of the current study. Protective mechanisms reducing peroxisomal H_2_O_2_ could include stimulation of pathways that inhibit the β‐oxidation of palmitic acid that generates the H_2_O_2_ or pathways that detoxify the H_2_O_2_ so produced. Pancreatic β cells are particularly vulnerable to oxidative stress due to low basal activity of protective pathways that lower intracellular ROS levels, such as low expression of the antioxidant enzymes superoxide dismutase, which catalyses the conversion of superoxide radicals into H_2_O_2_, and of catalase and glutathione peroxidase, both of which eliminate H_2_O_2_.^30^, ^31^, ^32^ There is evidence that expression of these enzymes can be induced under conditions of hyperglycaemia and by exposure to insulinotropic agents in wild‐type murine islets and cell lines.^36^, ^37^

A second important finding of the current study is that C‐peptide increases catalase expression and that the presence of both palmitic acid and C‐peptide have an additive effect relative to either alone. The highest expression detected was with 200 μmol/L palmitic acid and 5 nmol/L or with 200 μmol/L palmitic acid and 10 nmol/L C‐peptide, compared with just 10 nmol/L C‐peptide alone or 200 μmol/L palmitic acid alone. These results provide evidence that C‐peptide has autocrine protective capabilities in peroxisomes against excess palmitic acid‐induced ROS through a catalase mechanism. Thus, C‐peptide antioxidant capability is evidently not limited to inhibiting ROS generation by only acting on plasma membrane‐associated NADPH‐dependent oxidase or by improving mitochondrial respiration,^10^, ^11^, ^12^, ^13^ but extends to the stimulation of pathways that decrease toxic levels of H_2_O_2_ in peroxisomes where ROS does the most damage in β cells.^28^

Our results provide evidence for a model featuring novel organellar mechanisms involving activation of pathways that increase catalase expression levels. Taken together, our results provide further evidence suggesting that C‐peptide functions beyond its role for proper folding and disulphide bond formation of the A and the B chains of insulin within the β cell. These findings support models in which C‐peptide has acquired physiological roles that occur after its secretion from β cells and functions to provide antioxidant actions.^17^, ^18^, ^38^ Here, we consider the nature and components of the pathways resulting in secreted C‐peptide protecting β cells. C‐peptide binds to its receptor which could be GPCR146.^39^ Signalling at the β cell surface membrane or along its endocytic itinerary,^40^ the C‐peptide/GPCR complex presumably activates one or more cytosolic factors that elevate peroxisome proliferator activator (PPAR)‐γ levels in the cytosol, nucleus, or both cellular compartments, as shown in kidney or in lung epithelial cells.^41^, ^42^, ^43^ PPAR‐γ is a member of the nuclear receptor superfamily of ligand‐activated transcription factors, that is known to transcriptionally regulate catalase expression.^44^ The increase in PPAR‐γ could be achieved by C‐peptide signalling enhanced synthesis via transcription or translation or slowed degradation. The increase in PPAR‐γ might also be accompanied by an increase in its phosphorylation, which could increase catalase gene transcription leading to increased catalase, and consequently the observed attenuation of peroxisomal H_2_O_2_ levels in the setting of glucolipotoxicity. These underlying intracellular mechanisms of C‐peptide antioxidant activity against glucolipotoxicity are under investigation.

The results indicating that C‐peptide induces catalase expression and reduces peroxisomal peroxide levels in β cells do not in any way exclude other components of the peroxisomal antioxidant defence pathways being involved. In mammalian cells, several other ROS detoxifying enzymes are known to contribute to peroxisomal redox balance, which include superoxide dismutases, peroxiredoxins, glutathione S‐transferases, and epoxide hydrolases.^29^ It will be important to test whether C‐peptide displays any effects on superoxide dismutase as well as other antioxidant enzymes in addition to catalase as part of the mechanisms of protecting β cells. The findings support models in which C‐peptide stimulates antioxidant pathways, including those that functionally protect against the oxidative stress consequent to excess long‐chain fatty acid catabolism in peroxisomes. These antioxidant pathways raise the question whether similar mechanisms of C‐peptide protection in peroxisomes are active in other cell types known to be protected by C‐peptide, such as those of the endothelium and smooth muscle.

Given that C‐peptide is both made by and protects β cells, changes in C‐peptide levels, up or down, will have effects amplified by its autocrine action or lack thereof. Less secreted C‐peptide will result in less protection from oxidative stress which, depending on the oxidative load, will put β cells at risk of apoptosis, resulting in less secreted C‐peptide, less protection, and so forth in a downward spiral. Accordingly, more C‐peptide will result in more protection from oxidative stress and decreased apoptosis resulting in greater numbers of β cells surviving the oxidative threat in the setting of glucolipotoxicity. The autocrine implications of the model logically extends to therapeutic increases in C‐peptide, which would be predicted to decrease β cell oxidative stress and death by apoptosis, as shown here, in the setting of not only diabetes but also, possibly, obesity in the absence of diabetes, and thereby slow the loss of functional β cell mass. These results and observations provide further support to test for and develop co‐replacement C‐peptide with insulin therapy clinically. Interestingly, there is a general positive association between C‐peptide levels and the protection of β cell functional mass year after year of T1D.^2^, ^3^, ^4^, ^5^, ^6^ Also, consistent with the present findings, there is a negative association between C‐peptide levels and the rate of development of diabetic complications in T1D.^45^, ^46^, ^47^, ^48^, ^49^, ^50^ What is lacking in therapeutic interventions might be simply adding back more than part of what was lost in T1D, not only the insulin but the C‐peptide as well. Given that glucolipotoxicity is a shared setting of diabetes in general, these developments appear to apply to T2D as well as T1D subjects.

## CONFLICT OF INTEREST

The authors declare that there is no conflict of interest associated with this manuscript.

## AUTHORS’ CONTRIBUTION

PD and PL conceived and designed the experiments. PL, ND, RT, performed the in vitro experiments, analysed the data, and wrote different sections of the manuscript. DS, CW, and SW performed the immunohistochemistry and microscope study analysis on peroxisomes. All authors read and have approved the final manuscript.

## ETHICS STATEMENT

There is no ethics statement for this manuscript.

## Data Availability

The data that support the findings of this study are available from the corresponding author upon reasonable request.
